# Stress resilience and engagement enhancement under the usage of Igusa (*Juncus effusus* L. var. *decipiens* Buchen.) mat: a crossover study

**DOI:** 10.1038/s41598-026-47520-8

**Published:** 2026-09-08

**Authors:** Fadilla Zennifa, Yanli Xu, Akiko Isa, Erika Tomimatsu, Ziyou Xu, Yousuke Konno, Masataka Ikegami, Kuniyoshi Shimizu

**Affiliations:** 1 Department of Agro‑Environmental Sciences, Faculty of Agriculture, Kyushu University, 744 Motooka, Nishi‑ku, Fukuoka, 8190395 Japan; 2 Kyushu University Institute of Asian and Oceanian Studies, Kyushu University, 744 Motooka, Nishi-ku, Fukuoka, 8190395 Japan; 3 Ikehiko Corporation, 1052 Miyamatsu, Oki-machi, Mizuma-gun, Fukuoka, 830-0424 Japan

**Keywords:** Natural product, Stress resilience, Igusa, Electroencephalogram, Engagement, Psychophysiology

## Abstract

The world is changing rapidly, so humans need to adapt quickly to the stress they face. Various efforts have been made to strengthen resilience to stress, one of which is by promoting the use of natural materials. In the past, Igusa (*Juncus effusus* L. var. *decipiens* Buchen.) was one of the natural materials used every day in Japan for home interiors. However, the usage of this product in living environments has not been confirmed yet. To study the effects of igusa on daily life, we conducted a crossover experiment in which we imitated a living space using igusa mats and unscented polypropylene mats (control mats). During the experiment, the electrical activity of the brain, psychological changes and behavioral performance during a visual stimulus discrimination task were recorded along with post-analysis of the material emitted by those mats. The results of this study confirm that the volatiles emitted from the Igusa mat enhance the engagement level and stress resilience during the presented task. Additionally, the room with an Igusa mat showed ability to keep the room humidity stable. This experiment has been registered with the UMIN Clinical Trials Registry under the ID: UMIN000053368 on 19/01/2024.

**Supplementary Information:**

The online version contains supplementary material available at https://doi.org/10.1038/s41598-026-47520-8.

## Introduction

Natural products play an important role in supporting human well-being. In Japanese culture there are various natural products that been used for housing material such as Japanese cedar wood (Sugi)^1^, and Igusa. Igusa (*Juncus effusus* L. var. *decipiens* Buchen.) has been used in Japanese residences as a flooring material since before the nineteenth century. Because Igusa is traditionally believed to offer several health benefits^2^, it is important to provide scientific evidence regarding its potential effects on human psychophysiological changes on living space. For this reason, we examined Igusa in mat form. As a comparison, we used unscented polypropylene mat as control because this mat does not emit any identifiable aroma. Understanding the effects of Igusa mats within indoor living spaces may contribute to promoting human wellbeing. Wellbeing has become an increasingly important concern in modern society, where rapid daily demands can reduce individuals’ capacity for sustained engagement and increase their susceptibility to stress. These societal changes highlight the need to identify environmental factors that can support engagement and resilience in everyday life. Environmental factor and its effect to human brain has been investigated in previous study by Nakashima et al.^3^, investigated the effect of Japanese cedarwood on visual processing in human brain by showing the result of 18 participants. That study showed that Japanese cedarwood produced larger occipital negativities in humans. Regarding the correlation between environmental factors and wellbeing, stress resilience remains an aspect that has been relatively underinvestigated. Stress resilience is characterized by an initial response to a stressor followed by a recovery toward baseline levels^4–6^. Therefore, it is a process that unfolds over time. Identifying strategies that can enhance engagement and strengthen stress resilience may provide meaningful benefits for supporting overall wellbeing. In this experiment, electroencephalogram (EEG) is used to monitor the brain electrical activity. EEG is an appropriate measurement tool not only because it is non‑invasive and poses no known risks to participants, but also because it provides superior temporal resolution compared with other neuroimaging techniques, such as functional magnetic resonance imaging (fMRI)^7^. We emphasize the exploration of this olfactory stimulation on the changes of brain activities because the aroma after entering the olfactory bulb then will be travelled to the brain regions that process emotions and memory. The aroma compounds of the Igusa mat were analyzed using Gas Chromatography—Mass spectrometry (GC-MS). Three analytical techniques were employed in combination: Thermal Desorption Unit (TDU)-GC-MS, solvent extraction-GC-MS, and Solid Phase Microextraction (SPME)-GC-MS. As these methods differ in sampling approach, sample pretreatment, and injection technique, their combined use enabled the detection of a wider range of aroma compounds and allowed for a more accurate characterization of the overall profile of volatile constituents present in the Igusa mat. In this study, we analysed electrical brain activity based on its frequency. Theta was categorized as a signal in the 4–8 Hz range, alpha as a signal in the 8–13 Hz range, and beta as a signal in the 13–30 Hz range^8^. Gamma was categorized as a signal in the 30–59 Hz range. To reduce inter‑individual variability, relative power ratios were used. We also computed the β/(α + θ) engagement index, which has been widely applied to evaluate mental effort, mental attention investment, alertness, and task engagement^9,10^. The psychological states were assessed using Visual Analogue Scale (VAS) and Profile of Mood States Second Edition (POMS 2). Because a comfortable indoor environment can influence human psychophysiological activities^11–15^, we additionally investigated the effect of Igusa mat toward humidity, temperature and carbon dioxide (CO_2_) in the experimental room.

## Results

### Chemical analysis of the Igusa mat using GC-MS

#### Air sampling and volatile organic compounds (VOCs) analysis in the experimental room

**Fig. 1:**
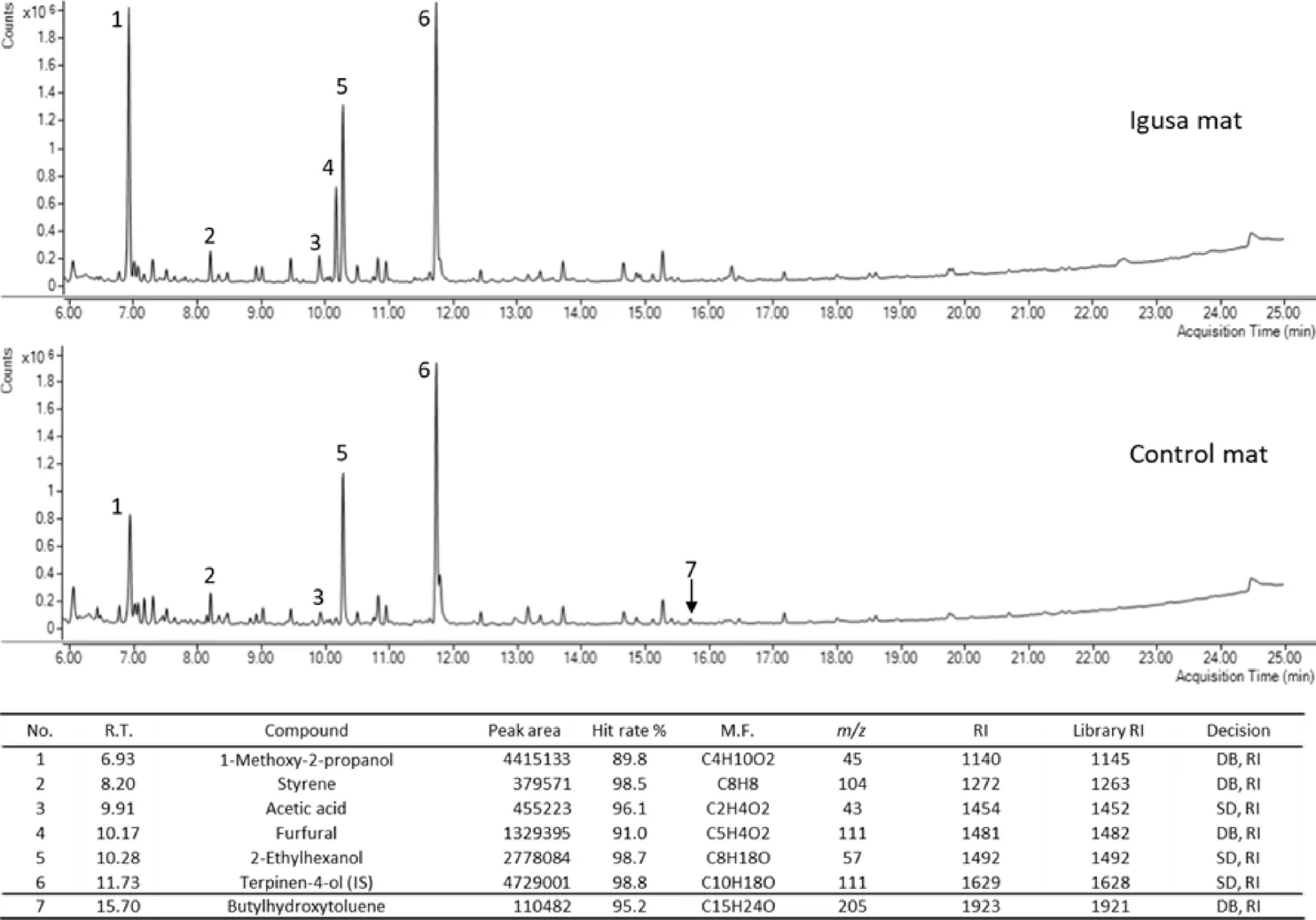
Chromatograms and tentatively identified compounds obtained by the TDU-GC–MS method. M.F. means molecular formula. DB means comparison with the database, RI means retention index, and SD means comparison of the retention time and mass with the standard substance, respectively.

Air samples were collected from the experimental room that contained either an Igusa mat or a control mat, and their volatile components were analyzed and compared using TDU-GC-MS analysis. Figure 1 shows a representative chromatogram obtained by TDU-GC-MS analysis and the identified compounds. As shown in Fig. 1, furfural (Peak No.4) was detected in the Igusa mat but not in the control mat, whereas butylhydroxytoluene (Peak No.7, BHT) was detected in the control mat but not in the Igusa mat. The furfural detected in the Igusa mat is presumed to be a VOC generated through thermal decomposition and dehydration reactions of pentoses (e.g., xylose, arabinose) derived from hemicellulose contained in Igusa, either during heat treatment in the processing stage or due to environmental factors such as temperature, humidity, and microbial activity during storage^16^. The BHT detected in the control mat is considered to be a commonly used additive that functions as an antioxidant and stabilizer in various chemical products^17^, and is presumed to have been intentionally added during the production of the polypropylene-based control mat.

#### Analysis of the Igusa mats using solvent extraction-GC-MS method

**Fig. 2:**
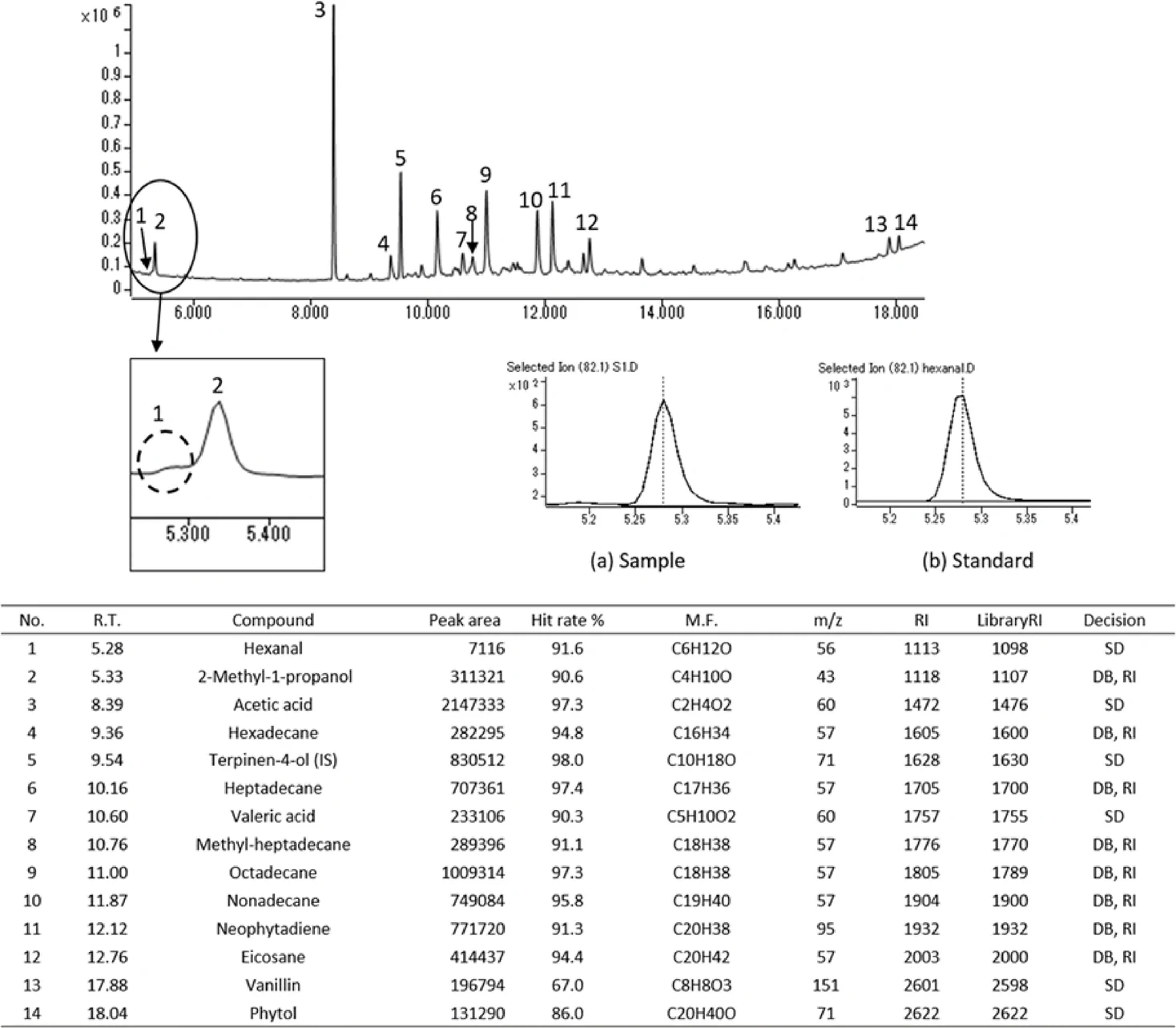
Chromatograms and tentatively identified compounds obtained by the solvent extraction-GC–MS method. M.F. means molecular formula. DB means comparison with the database, RI means retention index, and SD means comparison of the retention time and mass with the standard substance, respectively. The area enclosed by the dotted circle shows an enlarged view of the hexanal peak. Selected ion chromatogram (m/z 82) showing hexanal peaks: (**a**) Igusa sample, (**b**) standard compound.

The results of the analysis conducted after solvent extraction of the Igusa mat, which had been cut into 2–3 mm pieces, are shown in Fig. 2. Detailed examination of the detected peaks revealed that hexanal (Peak No. 1) was identified at a retention time of 5.28 min. The peak corresponding to hexanal is highlighted with a circle in the enlarged section of Fig. 2. In addition, the selected ion chromatogram for *m/z* 82 shows the hexanal peak for (a) the sample and (b) the standard compound. Hexanal is a characteristic aroma compound found in Igusa mat^18^, and is associated with a “green” or “grassy” scent.

Other characteristic aroma compounds contained in Igusa—acetic acid (Peak No. 3), valeric acid (Peak No. 7), and vanillin (Peak No. 13)—were also detected. Quantitative analysis showed that the concentrations of hexanal, acetic acid, valeric acid, and vanillin were 1.68, 228, 2.66, and 7.85 µg/g, respectively. However, the value for hexanal was below the quantification limit and is therefore considered a reference value. Among the compounds not subjected to quantitative analysis, neophytadiene (Peak No. 11) and phytol (Peak No. 14) were identified as Igusa-derived constituents, along with several other hydrocarbons (Peak No. 4, 6, 8, 9, 10, and 12).

#### Analysis of Igusa mats using SPME-GC-MS method

**Fig. 3:**
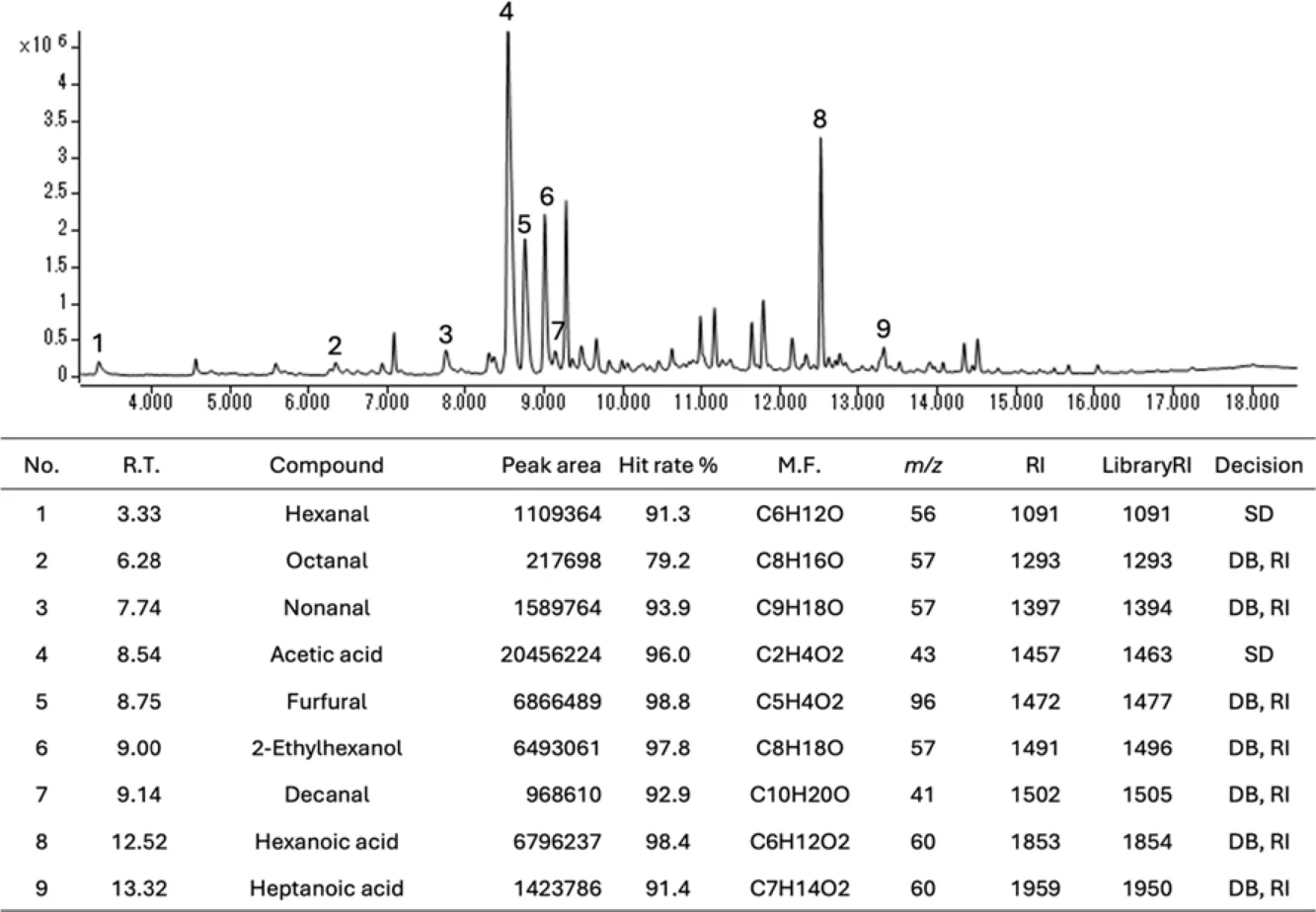
Chromatograms and tentatively identified compounds obtained by the SPME–GC–MS method. M.F. means molecular formula. DB means comparison with the database, RI means retention index, and SD means comparison of the retention time and mass with the standard substance, r*espectively.*

Figure 3 shows the chromatogram obtained by the SPME-GC-MS method along with the information on the estimated compounds. In SPME-GC-MS analysis, peaks originating from volatile compounds released from the fiber coating material—such as siloxanes and other polymer-derived substances—are often detected. Therefore, it is essential to carefully distinguish these background signals from sample-derived compounds. Peaks presumed to be fiber-derived were detected at retention times of 4.56, 7.09, 9.28, 9.47, 10.98, and 12.15 min, and were excluded from compound estimation. As can be seen in Fig. 3, hexanal (Peak No. 1) was clearly detected using the SPME-GC-MS method. Several aldehydes known to contribute to the aroma were also tentatively identified, including octanal (Peak No. 2), nonanal (Peak No. 3), furfural (Peak No. 5), and decanal (Peak No. 7).

#### Room environment analysis under different mats

**Fig. 4:**
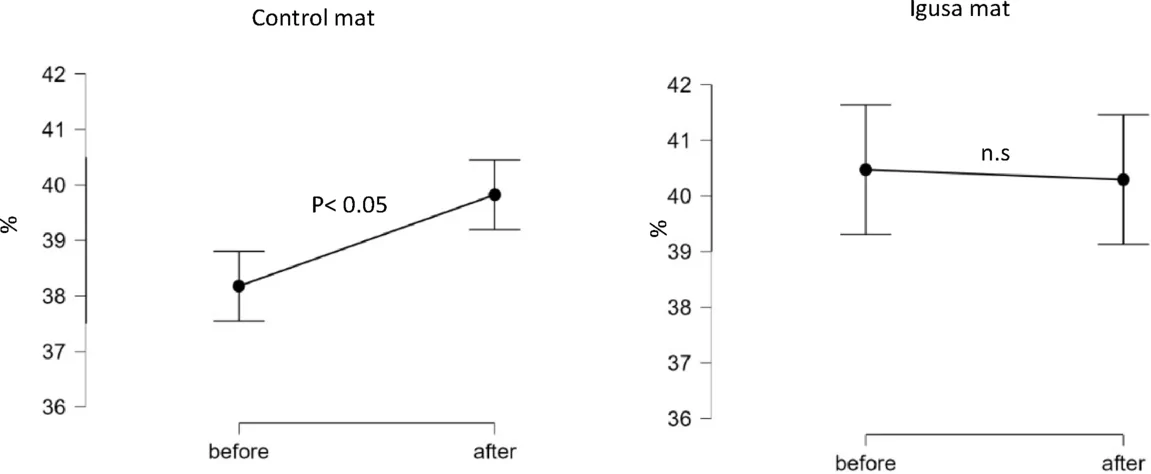
humidity under control mat and Igusa mat.

Humidity measurements taken before and after the experiment (Fig. 4) showed that the Igusa mat condition exhibited more stable humidity levels (before: 40.471 ± 7.409%; after: 40.294 ± 7.647%; *t*(16) = 0.227, *p* = 0.823, effect size = 0.055) compared with the control mat condition (before: 38.176 ± 8.516%; after: 39.824 ± 7.732%; *t*(16) = –3.926, *p* = 0.001, effect size = –0.952). In addition, the change in humidity (after – before) was evaluated for both conditions. The control mat showed a mean increase of 1.647 ± 1.73%, whereas the Igusa mat showed a mean change of –0.176 ± 3.206%. This difference was statistically significant (*z* = 2.016, *p* = 0.046, effect size = 0.592).

The change in temperature (after—before) did not differ significantly between the control mat (1.629 ± 0.680) and the Igusa mat (1.682 ± 0.862) conditions (*z* = –0.355; *p* = 0.740; effect size = –0.098). Similarly, the change in CO₂ concentration did not show a significant difference between the control mat (512.000 ± 170.765 ppm) and the Igusa mat (456.294 ± 157.204 ppm) conditions (*z* = 1.965; *p* = 0.051). However, a tendency toward lower CO₂ concentrations was observed in the Igusa mat condition.

#### Psychological evaluation (questionnaire test)

1. Participant Visual Analogue Scale (VAS)

Using VAS, perceptions of room aroma were quantified with 26 descriptors capturing fragrance characteristics. Compared with the control mat condition, the Igusa mat condition received higher ratings for a stronger aroma, greater likability, greater astringency, greater fragrancy, and greater relaxation, and lower ratings for “modern” but higher ratings for “antique,” as summarized in Table 1 (Wilcoxon signed‑rank test).

**Table 1:** Visual analogue scale on the impression of the aroma in the room.

Items	Condition		p	Effect size
	Igusa mat, N = 17	Control mat, N = 17		
Strength	71.0 ± 12.6	36.1 ± 25.5	**0.001**	− 0.902
Like	72.5 ± 12.0	58.9 ± 12.4	**0.008**	− 0.765
Bitter	35.2 ± 24.9	29.4 ± 22.2	0.320	− 0.281
Sweet	29.9 ± 21.3	22.9 ± 19.6	0.109	− 0.463
Astringent	62.8 ± 21.7	47.4 ± 23.0	**0.029**	− 0.608
Sour	26.5 ± 22.9	18.2 ± 18.4	0.116	− 0.486
Fragrant	71.6 ± 16.7	42.6 ± 24.8	**0.002**	− 0.869
Fresh	41.6 ± 18.1	39.3 ± 19.7	0.981	− 0.013
Relaxing	72.4 ± 10.0	64.9 ± 11.7	**0.044**	− 0.562
Plain	52.4 ± 16.9	58.2 ± 15.5	0.224	0.353
Comfortable	73.0 ± 11.7	64.5 ± 11.9	0.058	− 0.529
Refreshing	55.2 ± 17.9	54.4 ± 14.2	0.877	− 0.051
Clear	62.0 ± 15.7	59.5 ± 11.3	0.201	− 0.359
Elegant	61.9 ± 15.3	55.6 ± 16.6	0.266	− 0.324
Modern	24.3 ± 12.3	36.4 ± 18.5	**0.032**	0.618
Smelling	29.4 ± 18.2	19.5 ± 17.4	0.115	− 0.456
Nostalgic	71.6 ± 18.2	59.8 ± 21.7	0.059	− 0.544
Gentle	67.4 ± 12.1	61.4 ± 17.7	0.214	− 0.36
Calming	74.4 ± 9.5	67.1 ± 15.5	0.097	− 0.464
Gorgeous	27.4 ± 13.4	27.1 ± 16.0	0.906	0.039
Antique	76.8 ± 12.9	61.4 ± 18.6	**0.015**	− 0.68
Artificial	27.1 ± 12.1	34.6 ± 18.7	0.121	0.449
Rural	70.0 ± 18.7	60.6 ± 19.6	0.080	− 0.49
Natural	75.0 ± 12.0	62.3 ± 19.9	0.097	− 0.464
Heavy	36.3 ± 18.1	31.9 ± 18.1	0.313	− 0.294
Light	53.9 ± 17.0	51.5 ± 15.7	0.831	− 0.065

*Note*: Bold values indicate statistically significant differences between the Igusa mat and control mat conditions (*p* < 0.05).

2. Profile of Mood states 2 (POMS 2)

**Fig. 5:**
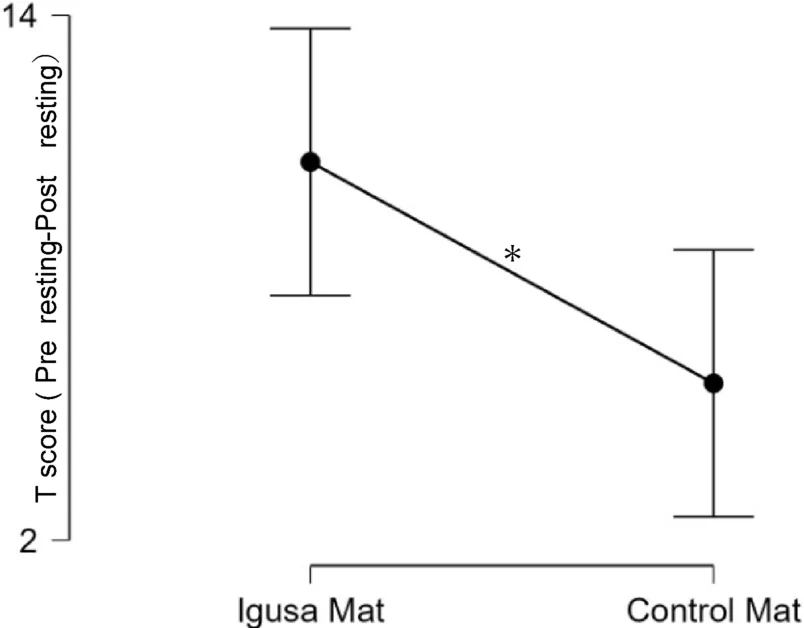
POMS (T score) under Igusa mat and control mat influence in tension-anxiety factor.

Seven mood scales and the associated total mood state score (TMD) were quantified using the short version of POMS2. Regarding the amount of change in each mood subscale, no significant difference was observed between the control and the Igusa mat conditions, except for the Tension–Anxiety (TA) subscale. For TA, the pre-post change under the Igusa mat condition (10.647 ± 10.350) was significantly grater than that under the control mat condition (5.588 ± 6.755)(*t* (16) = 2.488, *p* = 0.024, effect size = 0.603), as shown in Fig. 5.

3. Behavioural performance

**Fig. 6:**
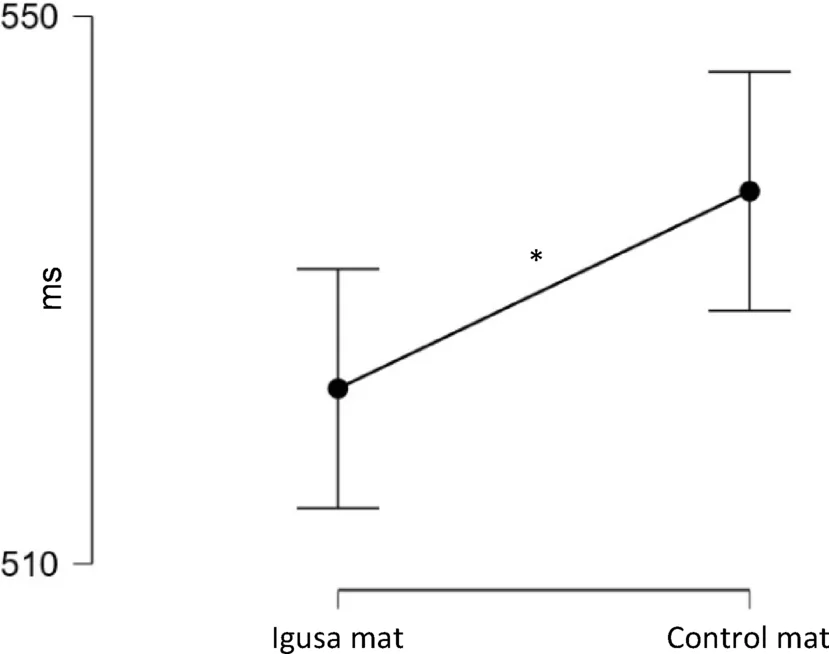
Reaction time.

We compared the two conditions between Igusa mat and control mat on participant reaction time and accuracy (hit accuracy). We found that reaction time was faster under Igusa mat condition (522.799 ± 55.943), compared with control condition (537.212 ± 45.604), *t*(16) = -2.476, *p* = 0.025, effect size = -0.600, as shown in Fig. 6, whereas there was no difference in hit accuracy percentage between the control and Igusa mat conditions (*z* = 0.207, *p* = 0.856, effect size = 0.059).

#### Electrical brain activities (physiological evaluation)

**Fig. 7:**
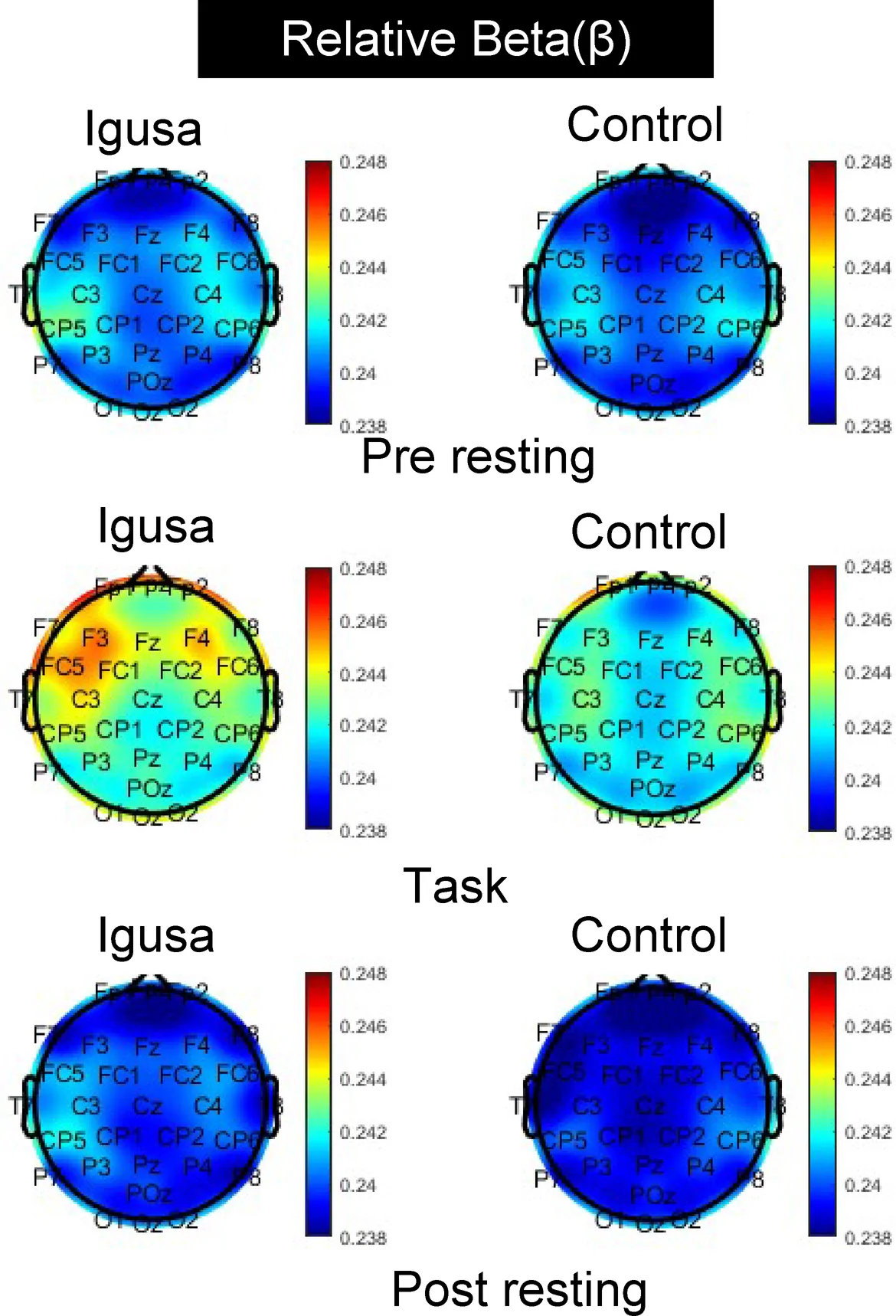
distribution of relative beta band.

**Fig. 8:**
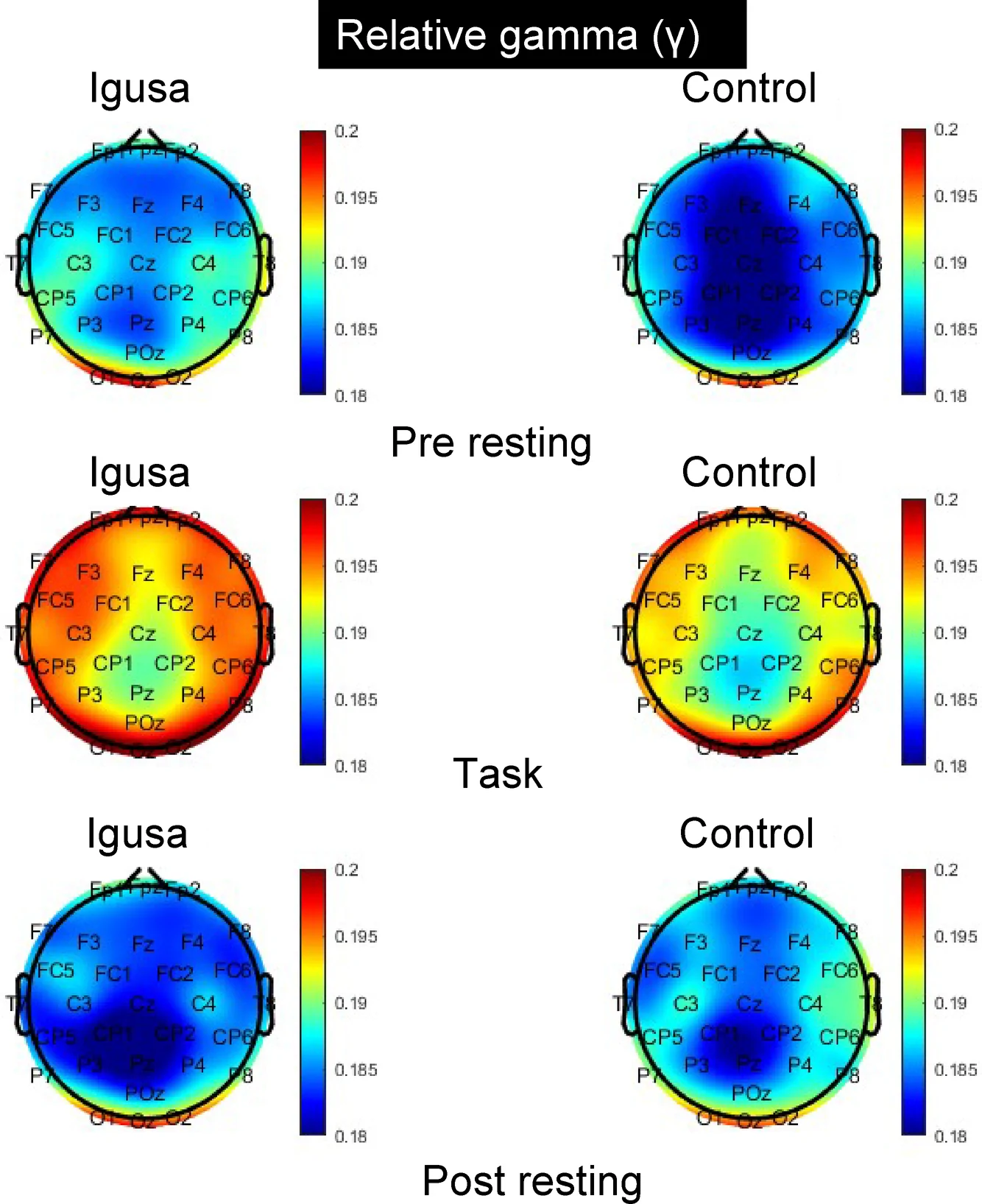
relative gamma band power distribution.

**Fig. 9:**
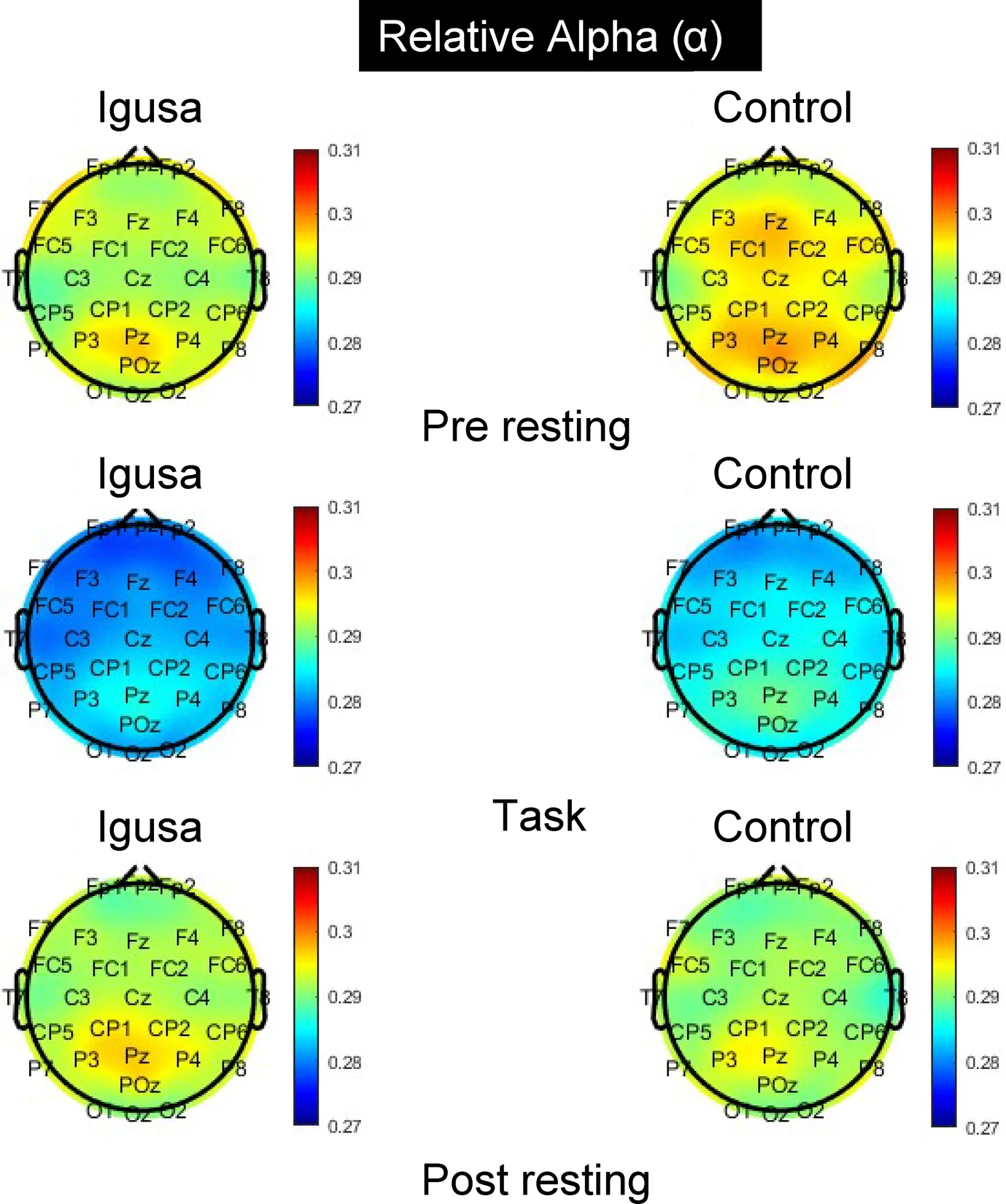
Relative alpha band power distribution.

**Fig. 10:**
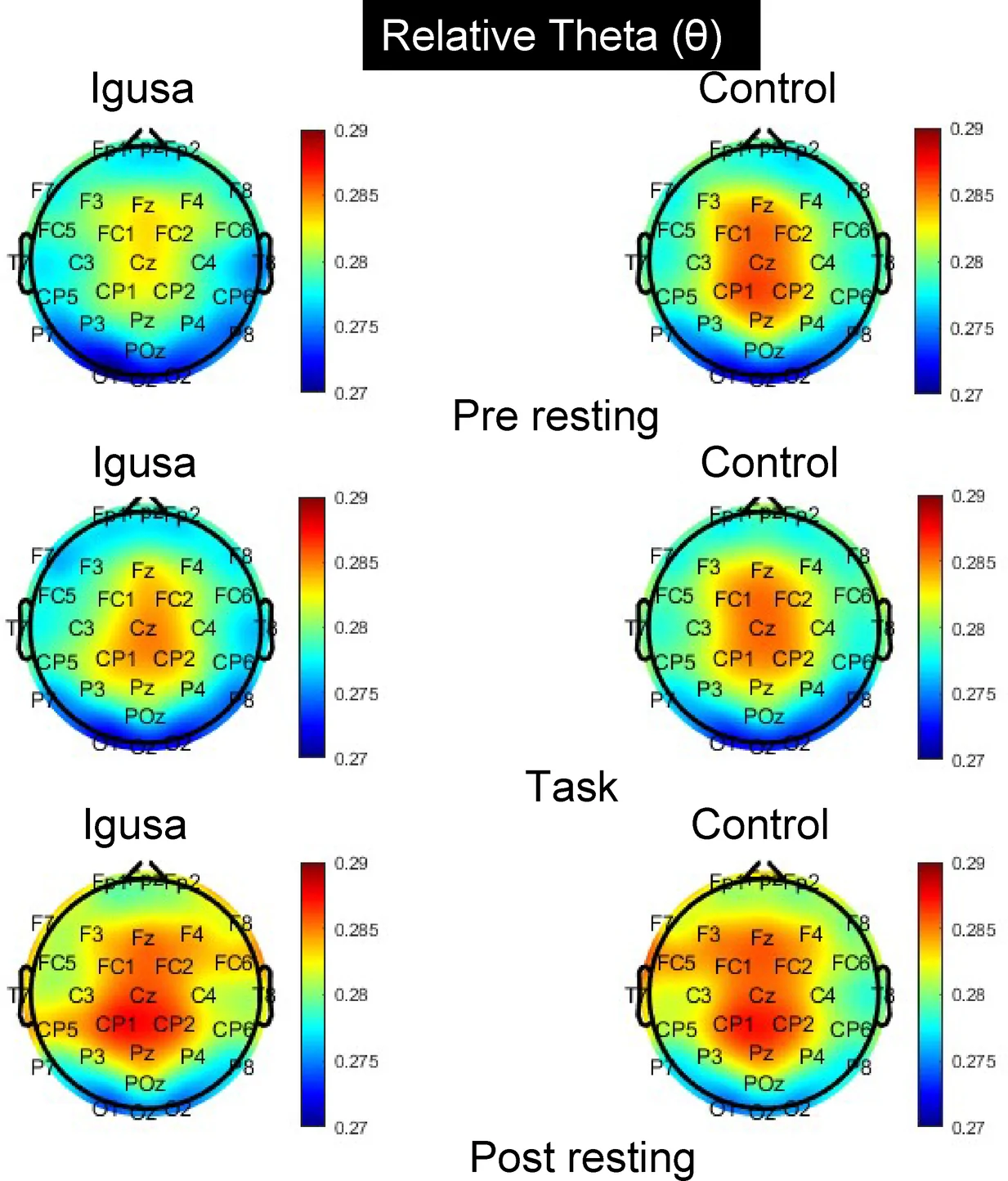
Relative theta band power distribution.

**Fig. 11:**
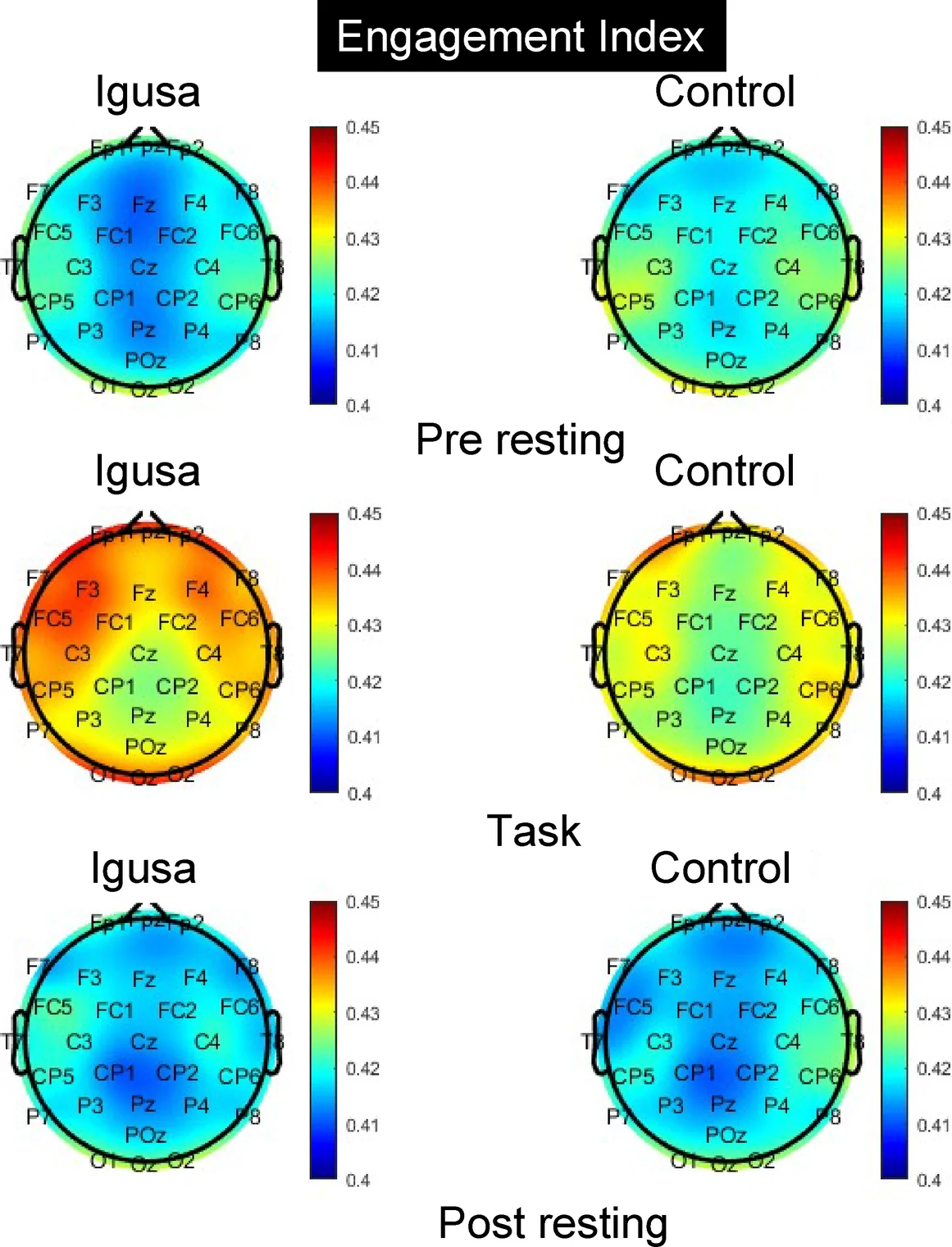
Engagement Index within two conditions during various situations.

In this experiment, we investigated the relative power distribution of EEG activities during resting and task performance. For relative beta band power, significant differences were observed between the Igusa mat and control mat conditions during the task, particularly in frontal area (Fpz, Fp2, F3, Fz, F4, FC5, FC1) and temporal area (T7). No differences were observed between the two conditions during the pre-resting period. After the task, additional differences were found at FC5, FC1, T7, CP5, and P3. The statistical comparisons for each electrode are summarized in Tables 2, 3, 4, 5, 6, 7, 8, 9, 10, and 11, where electrodes with significant differences are shown in bold. Changes in the relative power across frequency bands are illustrated in Figs. 7, 8, 9, 10, and 11.

**Table 2:** *p* value of relative beta band between Igusa mat and control mat.

Electrode placement	Preresting	Task	Postresting
Fp1	0.65	0.09	0.72
Fpz	0.98	**0.02**	0.49
Fp2	0.36	**0.04**	0.94
F7	0.94	0.08	0.43
F3	0.49	**0.01**	0.38
Fz	0.05	**0.01**	0.12
F4	0.08	**0.02**	0.41
F8	0.98	0.15	0.41
FC5	0.55	**0.04**	**0.04**
FC1	0.23	**0.01**	**0.04**
FC2	0.19	0.05	0.14
FC6	0.38	0.15	0.18
T7	0.23	**0.04**	**0.03**
C3	0.49	0.06	0.11
Cz	0.98	0.27	0.29
C4	0.94	0.49	0.83
T8	0.83	0.49	0.12
CP5	0.33	0.06	**0.02**
CP1	0.83	0.41	0.29
CP2	0.55	0.87	0.76
CP6	0.98	0.87	0.98
P7	0.29	0.79	0.59
P3	0.55	0.29	**0.01**
Pz	0.69	0.25	0.55
P4	0.62	0.83	0.38
P8	0.98	0.76	0.31
POz	0.09	0.08	0.49
O1	0.65	0.38	0.87
Oz	0.98	0.38	0.43
O2	0.59	0.19	0.98

*Wilcoxon test.

**Table 3:** Relative beta band multiple comparison.

Electrode placement	Igusa mat				Control mat			
	P*	Pre-task**	Pre-post**	Task-post**	P*	Pre-task**	Pre-post**	Task-post**
Fp1	**< 0.01**	0.01	1.00	0.02	**< 0.01**	0.02	1.00	0.01
Fpz	**< 0.01**	0.03	1.00	0.01	0.06	0.18	1.00	0.08
Fp2	**< 0.01**	0.01	1.00	0.02	0.08	0.51	1.00	0.08
F7	**< 0.01**	0.01	1.00	0.01	**0.02**	0.12	1.00	0.03
F3	**< 0.01**	< 0.01	1.00	0.01	0.11	0.18	1.00	0.26
Fz	**< 0.01**	0.01	1.00	0.02	**0.03**	0.05	1.00	0.08
F4	**0.02**	0.12	1.00	0.03	**0.03**	0.05	1.00	0.08
F8	**< 0.01**	0.01	1.00	0.01	**0.03**	0.08	1.00	0.05
FC5	**0.03**	0.05	1.00	0.08	**0.01**	0.69	0.26	0.01
FC1	**0.01**	0.01	0.69	0.26	0.08	0.51	1.00	0.08
FC2	0.06	0.08	1.00	0.18	**0.01**	0.05	1.00	0.02
FC6	**0.03**	0.08	1.00	0.05	**< 0.01**	0.05	1.00	< 0.01
T7	0.19	0.51	1.00	0.26	**0.03**	1.00	0.05	0.08
C3	**0.01**	0.26	0.69	0.01	**0.02**	1.00	0.12	0.03
Cz	0.05	0.05	1.00	0.26	**0.02**	0.51	0.51	0.02
C4	0.11	0.18	1.00	0.26	**0.01**	0.69	0.26	0.01
T8	**0.02**	0.51	0.51	0.02	0.05	0.26	1.00	0.05
CP5	0.33	0.51	1.00	0.69	**0.03**	1.00	0.05	0.08
CP1	0.33	0.69	1.00	0.51	**0.02**	1.00	0.12	0.03
CP2	0.19	0.26	1.00	0.51	0.33	1.00	0.69	0.51
CP6	0.16	1.00	0.18	0.69	**0.01**	0.69	0.26	0.01
P7	0.39	1.00	1.00	0.51	**0.01**	0.91	0.12	0.01
P3	0.10	0.37	1.00	0.12	**< 0.01**	0.51	0.18	< 0.01
Pz	**0.02**	0.03	1.00	0.12	**< 0.01**	0.26	0.26	< 0.01
P4	0.19	0.51	1.00	0.26	0.10	0.12	1.00	0.37
P8	0.05	0.26	1.00	0.05	**0.03**	0.03	0.91	0.37
POz	0.20	0.37	1.00	0.37	**0.01**	0.03	1.00	0.03
O1	**< 0.01**	0.02	1.00	0.01	**0.03**	0.08	1.00	0.05
Oz	**0.03**	0.08	1.00	0.05	0.05	0.26	1.00	0.05
O2	**< 0.01**	< 0.01	1.00	0.05	**0.03**	0.08	1.00	0.05

P*Friedman test.

P**Bonferroni.

**Table 4:** *p* value of relative gamma band between Igusa mat and control mat.

Electrode placement	Preresting	Task	Postresting
Fp1	0.83	0.91	0.83
Fpz	0.65	0.65	0.76
Fp2	0.94	0.72	0.79
F7	0.41	0.31	0.69
F3	0.36	0.49	0.69
Fz	0.41	0.72	0.65
F4	0.59	0.91	0.49
F8	0.87	0.52	0.38
FC5	0.76	0.49	0.62
FC1	0.08	0.29	0.83
FC2	0.36	0.31	0.65
FC6	0.98	0.65	0.18
T7	0.76	0.46	0.79
C3	0.21	0.25	0.41
Cz	0.12	0.46	0.49
C4	0.38	0.10	0.59
T8	0.41	0.19	0.43
CP5	0.41	0.59	0.09
CP1	0.07	0.27	0.55
CP2	0.08	0.33	0.65
CP6	0.76	0.91	0.62
P7	0.49	0.31	0.55
P3	0.46	0.69	0.59
Pz	0.12	0.31	0.72
P4	0.25	0.65	0.72
P8	0.23	0.25	0.41
POz	0.55	0.55	0.55
O1	0.33	0.59	0.76
Oz	0.55	0.72	0.83
O2	0.62	0.49	0.52

*Wilcoxon test.

**Table 5:** Relative gamma band multiple comparison.

Electrode placement	Igusa mat				Control mat			
	P*	Pre-task**	Pre-post**	Task-post**	P*	Pre-task**	Pre-post**	Task-post**
Fp1	**< 0.01**	0.01	1.00	0.02	**0.02**	0.03	1.00	0.12
Fpz	**< 0.01**	0.00	1.00	0.00	**0.01**	0.02	1.00	0.05
Fp2	**0.01**	0.12	0.91	0.01	**0.01**	0.05	1.00	0.02
F7	**0.02**	0.12	1.00	0.03	0.10	0.12	1.00	0.37
F3	**< 0.01**	0.02	1.00	< 0.01	**< 0.01**	< 0.01	0.08	0.69
Fz	**< 0.01**	0.01	1.00	0.01	**< 0.01**	< 0.01	0.37	0.12
F4	**< 0.01**	0.01	1.00	< 0.01	**0.02**	0.02	1.00	0.18
F8	**< 0.01**	0.03	1.00	0.01	0.06	0.08	1.00	0.18
FC5	**0.02**	0.02	1.00	0.18	**0.01**	0.01	0.91	0.12
FC1	**< 0.01**	0.05	1.00	< 0.01	**< 0.01**	< 0.01	0.02	1.00
FC2	**< 0.01**	0.03	1.00	0.01	**0.02**	0.02	0.18	1.00
FC6	**< 0.01**	0.05	1.00	< 0.01	**0.03**	0.03	0.37	0.91
T7	**0.01**	0.05	1.00	0.02	0.05	0.05	1.00	0.26
C3	**0.02**	0.18	1.00	0.02	0.10	0.12	0.37	1.00
Cz	**< 0.01**	0.26	0.26	< 0.01	** < 0.01**	< 0.01	0.12	0.37
C4	**0.03**	0.05	1.00	0.08	0.16	0.18	1.00	0.69
T8	0.05	0.26	1.00	0.05	0.10	0.12	0.37	1.00
CP5	**< 0.01**	0.69	0.08	< 0.01	0.10	0.12	1.00	0.37
CP1	**< 0.01**	0.51	0.18	< 0.01	**< 0.01**	0.00	0.18	0.51
CP2	**< 0.01**	0.26	0.26	< 0.01	**< 0.01**	< 0.01	0.51	0.18
CP6	**0.02**	0.18	1.00	0.02	**0.02**	0.03	1.00	0.12
P7	**< 0.01**	0.18	0.18	< 0.01	0.06	0.05	0.69	0.69
P3	**< 0.01**	0.37	0.12	< 0.01	**0.02**	0.02	1.00	0.18
Pz	**< 0.01**	0.18	0.05	< 0.01	**< 0.01**	< 0.01	0.69	0.08
P4	**0.01**	0.37	0.37	0.01	**< 0.01**	< 0.01	0.51	0.18
P8	**< 0.01**	0.08	0.69	< 0.01	**0.01**	0.01	0.91	0.12
POz	**< 0.01**	0.26	0.26	< 0.01	**0.02**	0.02	0.18	1.00
O1	**< 0.01**	0.08	0.26	< 0.01	**< 0.01**	< 0.01	0.69	0.08
Oz	**< 0.01**	0.26	0.26	< 0.01	**0.02**	0.02	1.00	0.18
O2	**< 0.01**	< 0.01	1.00	0.01	**< 0.01**	< 0.01	0.51	0.18

P*Friedman test.

P**Bonferroni.

**Table 6:** *p* value of relative alpha band between Igusa mat and control mat.

Electrode placement	Preresting	Task	Postresting
Fp1	0.55	0.43	0.91
Fpz	0.94	0.43	0.87
Fp2	0.98	0.31	0.94
F7	0.31	0.38	0.55
F3	0.83	0.21	0.25
Fz	0.21	0.14	0.87
F4	0.98	0.25	0.91
F8	0.94	0.52	0.79
FC5	0.94	0.21	0.36
FC1	0.09	0.08	0.91
FC2	0.31	0.08	0.76
FC6	0.59	0.25	0.55
T7	0.65	0.14	0.91
C3	0.38	0.14	0.62
Cz	0.16	0.16	0.98
C4	0.49	0.08	0.94
T8	0.76	0.29	0.21
CP5	0.23	0.11	0.46
CP1	0.43	0.12	0.46
CP2	0.33	0.16	0.91
CP6	0.91	0.59	0.79
P7	0.33	0.05	0.87
P3	0.59	0.10	0.91
Pz	0.69	0.06	0.76
P4	0.33	0.36	0.46
P8	0.25	0.23	0.52
POz	0.36	0.08	0.55
O1	0.62	0.08	0.76
Oz	0.46	0.19	0.55
O2	0.46	0.10	0.83

*Wilcoxon test.

**Table 7:** Multiple comparison relative alpha band.

Electrode placement	Igusa mat				Control mat			
	P*	Pre-task**	Pre-post**	Task-post**	P*	Pre-task**	Pre-post**	Task-post**
Fp1	**0.02**	0.03	1.00	0.12	**0.01**	0.01	0.91	0.12
Fpz	**< 0.01**	0.01	1.00	0.03	0.06	0.08	1.00	0.18
Fp2	**< 0.01**	< 0.01	1.00	0.01	**0.01**	0.02	1.00	0.05
F7	**< 0.01**	< 0.01	1.00	0.01	**0.03**	0.03	0.91	0.37
F3	**< 0.01**	0.01	1.00	< 0.01	**< 0.01**	< 0.01	0.12	0.37
Fz	**< 0.01**	0.01	1.00	0.02	**< 0.01**	< 0.01	0.69	0.08
F4	**< 0.01**	< 0.01	1.00	0.01	**< 0.01**	< 0.01	1.00	0.05
F8	**< 0.01**	0.01	1.00	0.01	0.05	0.05	1.00	0.26
FC5	**< 0.01**	0.01	1.00	0.02	**0.01**	0.01	0.37	0.37
FC1	**< 0.01**	0.01	1.00	0.01	** < 0.01**	< 0.01	0.37	0.12
FC2	**< 0.01**	0.01	1.00	0.02	**0.02**	0.02	1.00	0.18
FC6	**< 0.01**	< 0.01	1.00	0.01	**0.01**	0.01	0.69	0.26
T7	0.06	0.12	1.00	0.12	0.08	0.08	0.51	1.00
C3	**< 0.01**	0.01	1.00	< 0.01	**< 0.01**	< 0.01	0.05	0.51
Cz	**< 0.01**	0.05	1.00	< 0.01	**< 0.01**	< 0.01	0.18	0.51
C4	**0.01**	0.02	1.00	0.05	**0.03**	0.03	0.91	0.37
T8	**< 0.01**	0.03	1.00	0.01	0.10	0.12	0.37	1.00
CP5	**< 0.01**	0.02	0.69	< 0.01	**< 0.01**	< 0.01	0.51	0.18
CP1	**< 0.01**	0.02	1.00	< 0.01	**< 0.01**	< 0.01	0.37	0.12
CP2	**< 0.01**	0.08	0.69	< 0.01	**0.01**	0.01	0.37	0.37
CP6	**< 0.01**	0.01	1.00	< 0.01	0.10	0.12	1.00	0.37
P7	**< 0.01**	0.02	0.69	< 0.01	**< 0.01**	< 0.01	0.51	0.18
P3	**< 0.01**	0.00	1.00	< 0.01	**< 0.01**	< 0.01	1.00	0.05
Pz	**< 0.01**	0.01	1.00	< 0.01	**< 0.01**	< 0.01	0.51	0.18
P4	**< 0.01**	0.03	1.00	0.01	**< 0.01**	< 0.01	0.18	0.18
P8	**< 0.01**	0.01	1.00	< 0.01	**< 0.01**	< 0.01	0.69	0.08
POz	**< 0.01**	< 0.01	1.00	< 0.01	**< 0.01**	< 0.01	0.01	1.00
O1	**< 0.01**	< 0.01	1.00	< 0.01	**0.01**	0.01	0.69	0.26
Oz	**< 0.01**	< 0.01	1.00	0.01	**0.01**	0.01	0.26	0.69
O2	**< 0.01**	< 0.01	1.00	0.01	**0.01**	0.01	0.26	0.69

P*Friedman test.

P**Bonferroni.

**Table 8:** *p* value of relative theta band between Igusa mat and control mat.

Electrode placement	Preresting	Task	Postresting
Fp1	0.46	0.94	0.41
Fpz	0.62	0.46	0.52
Fp2	0.69	0.83	0.69
F7	0.79	0.16	0.87
F3	0.29	0.27	0.76
Fz	0.52	0.94	0.31
F4	0.76	0.83	0.59
F8	0.46	0.38	0.31
FC5	0.83	0.87	0.65
FC1	0.16	0.36	0.27
FC2	0.29	0.79	0.94
FC6	0.79	0.79	0.21
T7	0.69	0.49	0.76
C3	0.25	0.59	0.83
Cz	0.29	0.83	0.98
C4	0.36	0.62	0.83
T8	0.36	0.23	0.69
CP5	0.41	0.62	0.10
CP1	0.09	0.76	0.83
CP2	0.15	0.91	0.79
CP6	0.52	0.87	0.55
P7	0.41	0.94	0.72
P3	0.33	0.65	0.98
Pz	0.08	0.76	0.65
P4	0.19	0.72	0.94
P8	0.59	0.76	0.79
POz	0.46	0.98	0.87
O1	0.14	0.98	0.52
Oz	0.87	0.69	0.76
O2	0.94	0.72	0.79

*Wilcoxon test.

**Table 9:** Multiple comparison relative theta.

Electrode placement	Igusa mat				Control mat			
	P*	Pre-task**	Pre-post**	Task-post**	P*	Pre-task**	Pre-post**	Task-post**
Fp1	0.49	1.00	0.91	0.91	0.59	1.00	1.00	0.91
Fpz	0.39	1.00	0.51	1.00	1.00	1.00	1.00	1.00
Fp2	0.47	1.00	0.69	1.00	0.66	1.00	1.00	1.00
F7	0.10	1.00	0.12	0.37	0.47	1.00	0.69	1.00
F3	0.84	1.00	1.00	1.00	0.94	1.00	1.00	1.00
Fz	0.84	1.00	1.00	1.00	0.84	1.00	1.00	1.00
F4	0.29	1.00	0.91	0.37	0.79	1.00	1.00	1.00
F8	0.20	1.00	0.37	0.37	0.94	1.00	1.00	1.00
FC5	0.84	1.00	1.00	1.00	0.59	1.00	0.91	1.00
FC1	0.79	1.00	1.00	1.00	0.66	1.00	1.00	1.00
FC2	0.47	0.69	1.00	1.00	0.79	1.00	1.00	1.00
FC6	0.47	1.00	1.00	0.69	0.94	1.00	1.00	1.00
T7	0.11	1.00	0.26	0.18	0.66	1.00	1.00	1.00
C3	0.84	1.00	1.00	1.00	0.94	1.00	1.00	1.00
Cz	0.84	1.00	1.00	1.00	0.79	1.00	1.00	1.00
C4	0.94	1.00	1.00	1.00	0.94	1.00	1.00	1.00
T8	0.10	1.00	0.12	0.37	0.79	1.00	1.00	1.00
CP5	0.29	1.00	0.37	0.91	0.84	1.00	1.00	1.00
CP1	0.19	1.00	0.51	0.26	0.84	1.00	1.00	1.00
CP2	0.79	1.00	1.00	1.00	0.94	1.00	1.00	1.00
CP6	0.16	0.69	1.00	0.18	0.79	1.00	1.00	1.00
P7	0.10	1.00	0.12	0.37	0.94	1.00	1.00	1.00
P3	0.47	1.00	0.69	1.00	0.94	1.00	1.00	1.00
Pz	0.39	1.00	0.51	1.00	0.84	1.00	1.00	1.00
P4	0.66	1.00	1.00	1.00	0.66	1.00	1.00	1.00
P8	0.33	1.00	0.69	0.51	0.49	0.91	1.00	0.91
POz	0.19	1.00	0.51	0.26	0.79	1.00	1.00	1.00
O1	**0.02**	1.00	0.03	0.12	0.66	1.00	1.00	1.00
Oz	0.06	1.00	0.08	0.18	0.79	1.00	1.00	1.00
O2	0.66	1.00	1.00	1.00	0.84	1.00	1.00	1.00

P*Friedman test.

P**Bonferroni.

**Table 10:** *p* value of engagement index between igusa mat and control mat.

Electrode placement	Preresting	Task	Postresting
Fp1	0.75	0.34	0.75
Fpz	0.97	0.15	0.86
Fp2	0.94	0.12	0.83
F7	0.61	0.24	0.54
F3	0.40	0.07	0.80
Fz	0.08	0.06	0.56
F4	0.69	0.14	0.95
F8	0.85	0.35	0.24
FC5	0.94	0.13	0.17
FC1	0.05	0.05	0.34
FC2	0.19	0.09	0.55
FC6	0.68	0.40	0.70
T7	0.46	0.12	0.23
C3	0.20	0.19	0.92
Cz	0.23	0.18	0.75
C4	0.41	0.36	0.96
T8	0.67	0.31	0.07
CP5	0.18	0.10	0.87
CP1	0.23	0.13	0.92
CP2	0.32	0.39	0.82
CP6	0.75	0.89	0.36
P7	0.27	0.20	0.93
P3	0.33	0.24	0.68
Pz	0.22	0.15	0.86
P4	0.35	0.58	0.35
P8	0.34	0.64	0.90
POz	0.17	0.11	0.56
O1	0.40	0.28	0.92
Oz	0.63	0.32	0.86
O2	0.64	0.21	0.80

*Student test.

**Table 11:** Multiple comparison engagement index.

Electrode placement	Igusa mat				Control mat			
	P*	Pre-task**	Pre-post**	Task-post**	P*	pre-task**	Pre-post**	Task-post**
Fp1	**< 0.01**	0.02	1.00	0.01	**0.01**	0.03	1.00	0.03
Fpz	**< 0.01**	0.01	1.00	0.01	0.06	0.18	1.00	0.08
Fp2	**< 0.01**	0.01	1.00	< 0.01	**0.01**	0.05	1.00	0.02
F7	**< 0.01**	0.01	1.00	0.01	0.11	0.18	1.00	0.26
F3	**< 0.01**	< 0.01	1.00	< 0.01	**< 0.01**	< 0.01	0.51	0.05
Fz	**< 0.01**	< 0.01	1.00	< 0.01	**< 0.01**	< 0.01	0.91	0.03
F4	**< 0.01**	0.01	1.00	< 0.01	**0.01**	0.02	1.00	0.05
F8	**0.01**	0.08	1.00	0.01	**0.03**	0.08	1.00	0.05
FC5	0.05	0.05	1.00	0.26	**0.02**	0.12	1.00	0.03
FC1	**< 0.01**	0.01	1.00	0.02	**< 0.01**	0.00	1.00	0.05
FC2	**< 0.01**	0.01	1.00	< 0.01	**< 0.01**	0.02	1.00	0.01
FC6	**< 0.01**	0.01	1.00	< 0.01	**0.01**	0.01	0.37	0.37
T7	**< 0.01**	0.02	1.00	< 0.01	**0.02**	0.12	1.00	0.03
C3	0.05	0.26	1.00	0.05	**0.02**	0.03	1.00	0.12
Cz	**< 0.01**	0.03	0.91	< 0.01	**0.02**	0.02	1.00	0.18
C4	**0.01**	0.03	1.00	0.03	**0.03**	0.05	1.00	0.08
T8	**< 0.01**	0.01	1.00	< 0.01	0.06	0.08	1.00	0.18
CP5	**0.02**	0.51	0.51	0.02	**<** **0.01**	0.05	1.00	< 0.01
CP1	**< 0.01**	0.00	1.00	0.01	**0.03**	0.08	1.00	0.05
CP2	**0.01**	0.12	0.91	0.01	0.06	0.08	1.00	0.18
CP6	0.06	0.69	0.69	0.05	**0.03**	0.08	1.00	0.05
P7	**< 0.01**	0.12	0.37	< 0.01	**0.02**	0.12	1.00	0.03
P3	**0.02**	0.51	0.51	0.02	**0.01**	0.08	1.00	0.01
Pz	**< 0.01**	0.02	1.00	< 0.01	**< 0.01**	0.05	1.00	< 0.01
P4	**< 0.01**	0.18	0.51	< 0.01	**0.01**	0.01	1.00	0.08
P8	**0.01**	0.08	1.00	0.01	**< 0.01**	0.02	1.00	0.01
POz	**< 0.01**	0.01	0.91	< 0.01	**0.01**	0.03	1.00	0.03
O1	**< 0.01**	0.01	1.00	< 0.01	**0.02**	0.03	1.00	0.12
Oz	**0.01**	0.05	1.00	0.02	**0.01**	0.01	1.00	0.08
O2	**0.01**	0.01	1.00	0.08	**0.01**	0.01	1.00	0.08

P*Friedman test.

P**Bonferroni.

##### Engagement index calculation

## Discussion

Igusa material has been used in Japanese home interiors for centuries. The effects of Igusa on psychophysiological responses and indoor environmental conditions have not yet been thoroughly investigated in the scientific literature. Igusa mats are known to emit characteristic aromas and have the ability to absorb moisture, which helps stabilize indoor humidity levels compared with conditions without Igusa. Humidity is one of several factors that contribute to a comfortable indoor environment^11^. Our results showed that under Igusa mat condition, indoor humidity remained stable at approximately 40% between before and after task during stress induction exposure. Based on the POMS 2 results, participants showed greater resilience to tension-anxiety (TA) under the Igusa mat condition. In the POMS2 results, a significant difference was observed only in the Tension–Anxiety subscale. Therefore, the observed effect should be interpreted as specific to this stress-related dimension rather than a general improvement across all mood domains. The non-significant pre-post changes in alpha band activity during resting condition could also serve as an indicator of stress resilience. In addition, to characterize the volatile compounds emitted from the Igusa and control mat used in the experiment, we carried out GC–MS analysis on both materials. In this study, air sampling analysis conducted in the laboratory detected furfural and acetic acid as aromatic compounds presumed to be derived from Igusa. However, the air sampling analysis revealed that the concentration of aroma compounds derived from the Igusa mat was low, and numerous compounds originating from sources other than the Igusa mat were also detected, making it difficult to obtain sufficient information on the aroma profile. Therefore, a detailed analysis of the aroma compounds in the Igusa mat was conducted using a combination of solvent extraction and SPME methods, which identified the presence of hexanal, acetic acid, valeric acid, and vanillin. In addition to these compounds, furfural—detected by both TDU and SPME methods—and several aromatic aldehydes identified via SPME were presumed to contribute to the characteristic aroma of the Igusa mat. In contrast, analysis of the control mat using the TDU method detected only acetic acid as a volatile compound. Since acetic acid can also be detected from environmental sources, the results indicated that aroma compounds originating from the control mat were significantly fewer compared to those from the Igusa mat. The Igusa mat used in this experiment contained characteristic aroma compounds of Igusa; however, the concentration of hexanal was relatively low. The reduction in hexanal concentration was attributed to both thermal losses during manufacturing and subsequent volatilization and degradation during storage. In the analysis using the TDU method, the collection efficiency of target compounds may be compromised when their concentrations are low or when numerous volatile compounds coexist. Accordingly, the use of chemically selective adsorbents and the optimization of pretreatment conditions are considered essential for improving analytical performance.

Based on VAS results, participants preferred the aroma of Igusa mat over that of the control mat. Because Igusa has historically been used in Japanese living environments, some participants may have had prior familiarity with its scent. This factor may have influenced subjective perception and should be considered in future studies. This preference was also reflected in the gamma band activity. Relative gamma band power in this study showed significant differences under the igusa mat condition across widespread brain regions between the resting and task conditions. Gamma band activity is known to be associated with the presence of olfactory stimuli, a pattern that was also observed in our previous study^19^. On the other hand, under the control mat, several areas on the brain (F7, F8, T7, C3, C4, T8, CP5, P7) which are areas that correlate with the effect of the presence of aroma in the air related to relative gamma power did not show differences within 3 activities. Relative to the control mat, the Igusa mat was rated as producing a more relaxed and more “antique” impression. By contrast, the control mat was rated as “modern” than the Igusa mat. The aroma of Igusa mat was perceived as stronger and more astringent than that of the control mat. This astringency may be attributed to the presence of acetic acid and valeric acid among the Igusa derived volatiles. Consistent with this interpretation, increased relative beta band power was observed over left-hemisphere regions during the post resting condition. We also found that participants’ reaction time were faster in the Igusa mat condition than that in control condition. Attention is known to enhance response speed^20^, whereas mental fatigue and reaction time increase as task duration becomes longer^21^. Therefore, our results on reaction time suggest that the Igusa mat condition may help sustain attention over time in the presence of mental fatigue. This pattern was also evident in the relative beta band results, in which frontal regions showed higher relative power under the Igusa mat condition compared to the control mat. The engagement index showed higher values during task performance under the Igusa mat condition, particularly at electrode FC1, compared with the control mat condition. Based on these findings, the Igusa mat may be suitable for daily activities that require sustained task engagement. Although the sample size of the present study was relatively modest, the crossover design allowed each participant to serve as their own control, which increases statistical sensitivity. Future studies with larger samples would help further validate these findings. During the resting condition, no significant change was observed under the Igusa mat condition, whereas a significant decrease was observed under the control mat condition. In other words, the Igusa mat may help maintain resting state stability and, by extension, could support stress resilience compared with the control condition.

## Methods

### Ethical matters

This study evaluated the effects of Igusa mat use on human psychophysiological activities. This study was approved by the Ethics Committee of the Faculty of Agriculture, Kyushu University (Application number:23-010, Approval number: 115, Project title: Exploratory Study on the Functionality of Igusa Goza), Approval date: 27th December 2023. This experiment was registered in the UMIN Clinical Trials Registry (Register ID: UMIN000053368 on 19th January 2024). All measurements involving human participants were performed in accordance with the Declaration of Helsinki. Before participating, all participants were fully informed of the purpose and procedures of this study. Written informed consent was obtained from each participant, and participation was voluntary. Each participant received 4000 yen as compensation at the end of the experiment.

### Participants

Recruitment information for this experiment was posted on Kyushu University’s information bulletin board. Participants interested in this study were asked to register online using the QR code posted on the bulletin board. Prior to the experiment, 27 individuals had registered through the online participant registration form. Those registered participants were selected based on the following criteria:

1. Native Japanese speakers
2. Those who can refrain from wearing perfume or strongly scented hair products on the day of the experiment
3. Those who are comfortable in enclosed spaces
4. Non-smokers
5. Those who are not currently suffering from olfactory abnormalities due to a cold or other illness
6. Not undergoing treatment for psychosomatic or neurological disorders

**Fig. 12:**
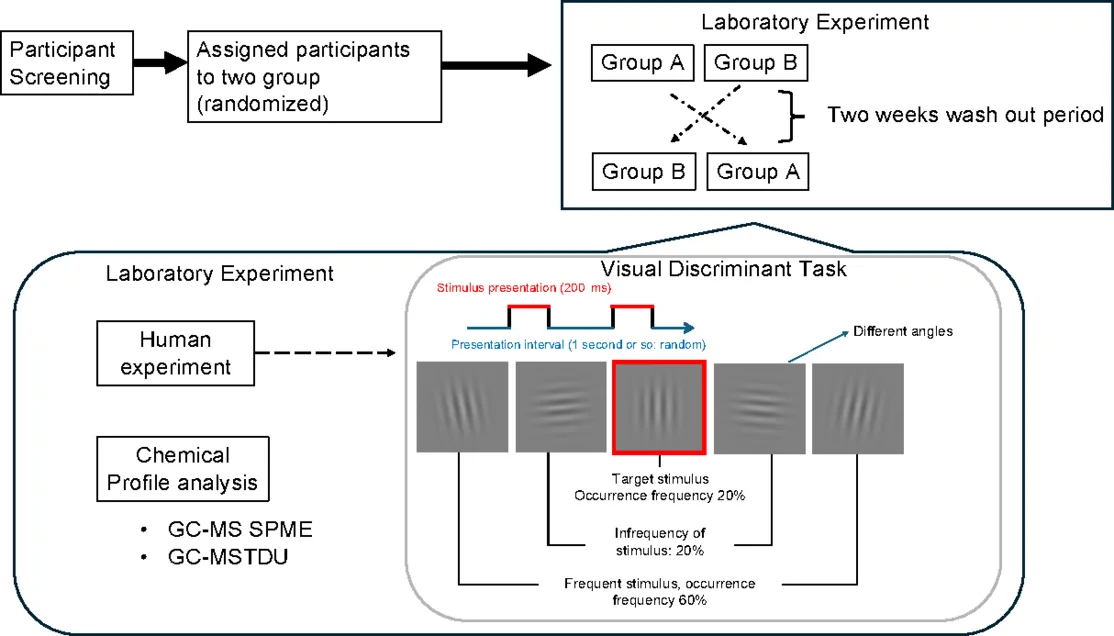
General clinical trial and chemical analysis experiment’s flow.

After screening process, 18 participants (10 males and 8 females) were included. One participant was excluded after withdrawing from the experiment midway for personal reason. The final sample consisted of 17 participants (10 males and 7 females) with a mean age of 21.53 ± 1.91 years old. The age distribution of the participants is provided in Supplementary Table S1. The study employed a randomized crossover design (Fig. 12). Participants were not informed which mat would be presented prior to each session. They were randomly assigned by computer to two groups of nine and took part in the study twice. In Group A, the control (polypropylene) mat was used in the first session and the Igusa mat on a separate day. In group B, the Igusa mat was used in the first session and the control (polypropylene) mat on the other day. The washout period for the preceding and subsequent test sessions was approximately 2 weeks. Experimental sessions were conducted within two fixed time windows (10:00–12:00 or 14:00–16:00). Each participant completed both the control and Igusa conditions on separate days within the same time window to minimize potential circadian influences on EEG and psychophysiological measures. The study was conducted from February 2^nd^ to March 21^st^, 2024.

### Experimental room and Igusa presentation

**Fig. 13:**
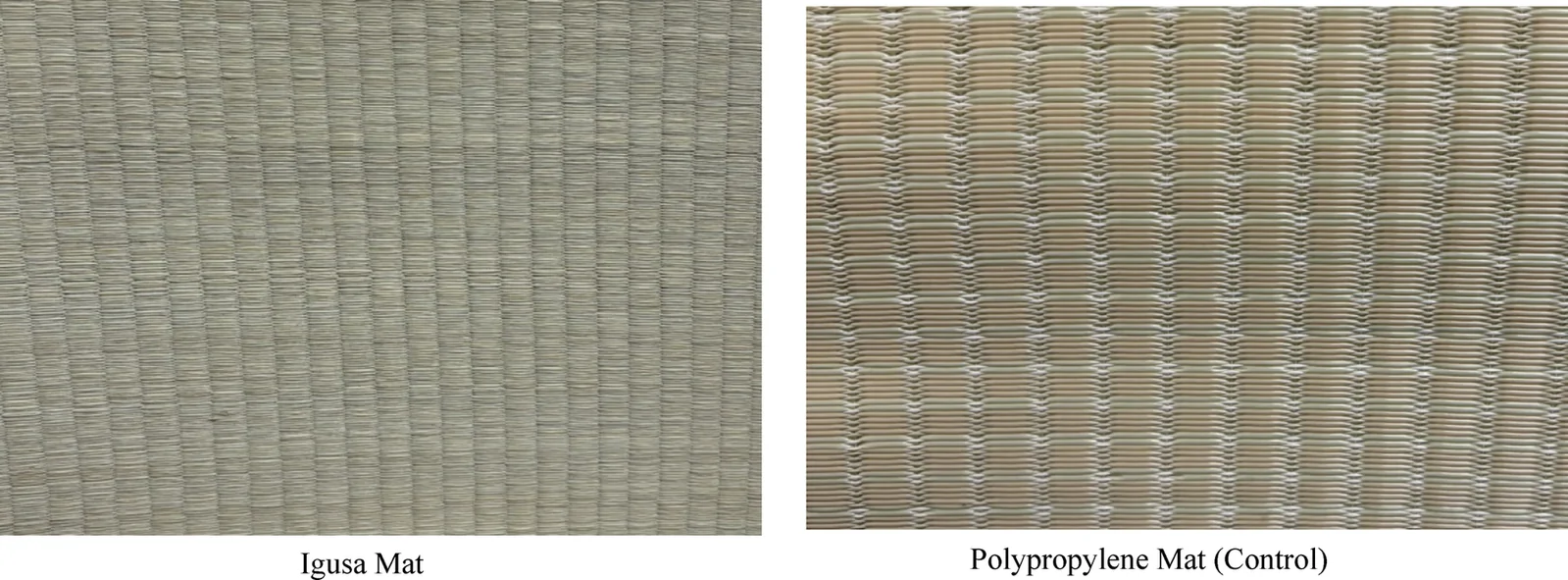
Igusa mat and polypropylene mat (control) ‘s appearance.

This experiment was conducted inside soundproof room (width 1,700 mm, depth 1,260 mm, height 1,965 mm, manufactured by Yamaha Corporation), hereinafter referred to as the laboratory, installed in Room 457 of West 5 building at Kyushu University. The test was conducted in a 4.2 m^3^ experimental space under conditions that minimized external auditory and visual stimulation. Temperature and humidity were monitored by using TR-72wf T&D Temperature and Humidity Data Logger (T&D Co., Ltd.), and carbon dioxide concentration was measured by testo 160 IAQ – Cloud monitoring logger (Testo SE & Co. KGaA). The Igusa mat and control mat (Fig. 13) were provided by Ikehiko Corporation. No ventilation fans were used during the test, and the doors closed and the light was off to eliminate the effects of external sound and light. Communication between outside room and inside laboratory was maintained via web camera (SwitchBot camera 3MP).

### Experimental procedure

**Fig. 14:**
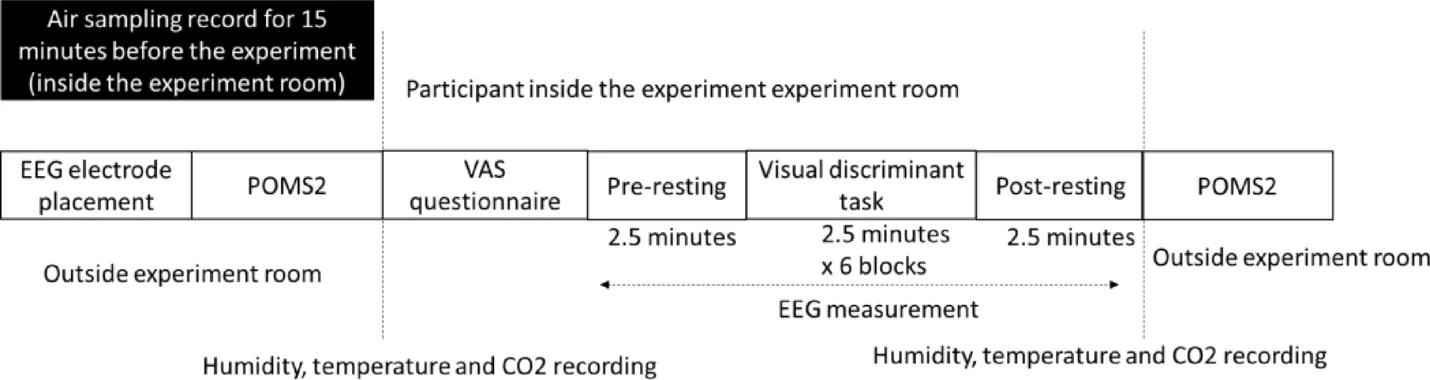
Experiment timeline during clinical trial.

To minimize odor contamination from clothing, participants changed into cotton laboratory garments provided by the investigators approximately 10 min before the start of the experiment. After neurophysiological sensors had been attached to the participants, they completed the POMS 2 questionnaire. Participants were then escorted to the laboratory and seated on the assigned mat. Prior to the visual discriminant task appearing, participants completed VAS questionnaire (the experimental flow is shown in Fig. 14). A 1.5‑min rest interval was provided between task blocks, during which participants were permitted to stretch to minimize fatigue.

### GC–MS analysis

**Fig. 15:**
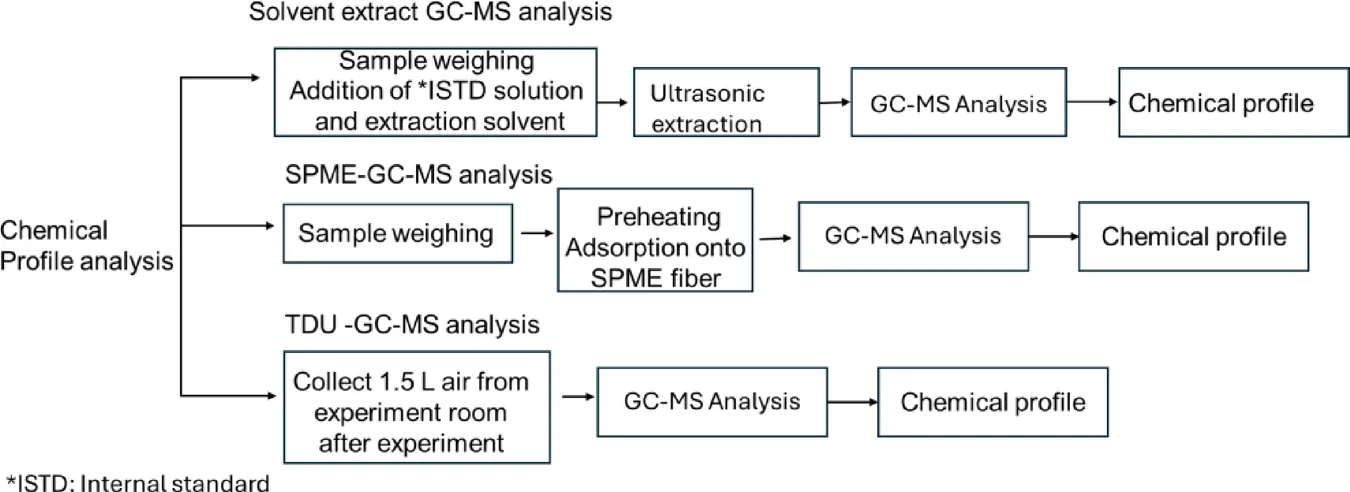
Workflow of the GC–MS chemical profiling analysis (solvent extraction, SPME, and TDU methods)

The procedures for air Sampling and volatile organic compounds (VOCs) analysis in the experimental room, together with the chemical profiling of materials by using gas chromatography–mass spectrometry system (GC–MS), are summarized in Fig. 15.

TDU-GC-MS method

Air pumps (mini pump MP-Σ30NⅡ; SIBATA, Tokyo) connected to sorbent tubes (Tenax TA; Gerstel, Linthicum, MD, USA) were installed in the experiment room immediately after the psychophysiological evaluation experiment, and were started simultaneously to collect VOCs (flow rate: 0.15 L/min, amount: 1.5 L, duration: 10 min). The sorbent tubes were thermally desorbed into a gas chromatography–mass spectrometry system (GC–MS; 7890A/5975C, Agilent Technologies, Santa Clara, CA, USA) via a thermal desorption unit (TDU) (Gerstel). The GC–MS was equipped with a DB-WAX column (30 m × 0.25 mm; 0.25 µm film thickness; Agilent Technologies). The oven temperature program was 60 ℃ for 3 min and increased to 150 ℃ at 20 ℃/min, then increased to 240 ℃ at 5 ℃/min and held at this temperature for 5 min. Helium was used as the carrier gas at a flow rate of 1.5 ml/min. The measurement was conducted using splitless injection and scan mode. A terpinen-4-ol-acetone solution (100 µL/L) was added as an internal standard (ISTD). Compounds in the air samples of the Igusa and control mats were tentatively identified by comparing the mass spectrum library (Wiley 9th and NIST17) and determined the Kovats retention index (RI) using Aroma Office software ver. 7.0 (Nishikawa Keisoku, Tokyo, Japan).

Chemical analysis of the Igusa mat.

For chemical analysis, the Igusa portion of the mat was excised and shredded into 2–3 mm fragments.

Solvent extraction-GC-MS method

Ethyl acetate (Concentration 5000; FUJIFILM Wako Pure Chemical Co., Ltd., Tokyo, Japan) was employed as the extraction solvent. As a moderately polar organic solvent commonly used for isolating plant-derived compounds, it enables efficient extraction of a wide spectrum of constituents. Hexanal (≥ 95% purity; FUJIFILM Wako Pure Chemical Co., Ltd.), acetic acid (glacial, 99.9985% purity; FUJIFILM Wako Pure Chemical Co., Ltd.), valeric acid (≥ 98% purity; Tokyo Chemical Industry Co., Ltd., Tokyo, Japan), and vanillin (≥ 98% purity; Tokyo Chemical Industry Co., Ltd.) were used as standard compounds. Terpinene-4-ol (≥ 90% purity; FUJIFILM Wako Pure Chemical Co., Ltd.) was used as the internal standard.

A 0.5 g sample of Igusa was mixed with 1 µL of an ethyl acetate solution of terpinen-4-ol (1.0 × 10^3^ mg/L, used as an internal standard) and 10 mL of ethyl acetate. After vortexing for 10 s, ultrasonic extraction was performed at 40 kHz for 20 min. The extract was then filtered through a 0.2 µm PTFE filter (PTFE-HI, Agilent Technologies), and 1 µL of the filtrate was injected into the GC–MS. The GC–MS analysis was carried out on a gas chromatograph (7890B, Agilent Technologies, CA, USA) coupled with an EI mass spectrometer model (5977B, Agilent Technologies). The GC was equipped with a HP-INNOWAX capillary column (Agilent Technologies); 30 m length, 0.25 mm diameter, 0.25 µm film thickness. The applied temperature program was started at 40℃ for 3 min, then rising to 140℃ at a rate of 20℃/min, then with 10℃/min. to 260℃ and kept for 10 min. The helium flow rate was 1.5 mL/min. The sample injection volume was 1 µL and operated to the splitless mode. Electron ionization mass spectra were collected at 70 eV ionization energy over an m/z scan range of 45–450 amu, using both Selected Ion Monitoring (SIM) and scan modes. The MS source temperature was set at 230℃.

Compounds in the Igusa sample were tentatively identified by comparing the mass spectrum library and determined the RI using Aroma Office software ver. 7.0.

Quantitative analysis was performed by constructing calibration curves using standard compounds of hexanal, acetic acid, valeric acid, and vanillin, and calculating the concentration values accordingly. SIM analysis was conducted using MassHunter software (Ver.10.1, Agilent Technologies). The calibration curves were prepared using the internal standard method. The concentration ranges were set at 0–5 µg/mL for hexanal, valeric acid, and vanillin, and 0–50 µg/mL for acetic acid.

SPME-GC-MS method

SPME–GC–MS analysis was carried out to investigate the volatile components of the Igusa sample. SPME fibers (50/30 µm, DVB/CAR/PDMS, gray, 20 mm) and holders used in this study were commercially available from Supelco (Bellefonte, PA, USA).

The SPME–GC–MS method was conducted using a headspace technique, in which volatile compounds were adsorbed onto a fiber and subsequently analyzed by GC–MS. An aliquot of 0.5 g was placed in a 20 mL vial and maintained at 60 ℃ for 30 min to allow thermal equilibration. After preheating, volatile compounds were adsorbed onto the fiber at 60℃ for an additional 30 min, followed by GC–MS analysis. Except for the temperature program, all analytical conditions including the GC–MS instrument and column were identical to those used in the solvent extraction GC–MS method. The temperature program was as follows: initial hold at 50 ℃ for 2 min, ramped at 10 ℃/min to 120 ℃, then at 15 ℃/min to 250℃, followed by a final hold at 250 ℃ for 13 min.

### Psychological evaluation

An overview of the two questionnaire tests conducted is shown below.

Visual Analog Scale (VAS)

Visual analogue scale (VAS) was used to investigate the impression of scent in room space. The VAS is a widely used assessment method for evaluating pain intensity and treatment efficacy. In recent years, it has been widely used in the medical field to measure health related quality of life (QOL). It has also been applied to measure post-sleep alertness, quality of life, anxiety, breathlessness, nausea, dyspnea, pruritus intensity, and attitude toward the environment. Furthermore, its usefulness has been reported in numerous applications in research on education, sound environments, and olfactory perception. In this study, the VAS was administered digitally using the Research Electronic Data Capture (REDCap) provided by Kyushu University Academic Research Organization (ARO) Next Generation Medical Center^22^.

In this test, the left end of the scale was labeled ‘‘no impression’’ (0), and the right end was labeled ‘‘impression’’ (100). Participants moved the slide according to their perceived impression. The evaluation items consisted of adjectives and verbs expressing fragrance impressions, and responses to a total of 21 items were estimated as a VAS score (%).

POMS2 (Profile of Mood States Second Edition)

The Japanese version of the Profile of Mood States Second Edition (POMS 2; abbreviated version; Kaneko Shobo), was used to evaluate mood states and compare the states before and after fragrance presentation. POMS 2 is a revised form of the original POMS and enables rapid assessment of both relatively stable mood states and transient emotional fluctuations. “Anger – Hostility (AH)”, “Confusion – Bewilderment (CB)”, “Depression – Depressed (DD)”, “Fatigue – Inertia (FI)”, “Tension – Anxiety (TA)”, “Vigor – Activity (VA)”, and “Friendliness (F)”. In addition, the Total Mood Disturbance (TMD) score provides a composite index representing overall negative mood. The same assessment procedure employed in our previous study^19^ was used in this investigation.

### Physiological evaluation

During EEG recording, cloth-like electromagnetic shields (Noi Cut Sheet 44,552, GE Healthcare Japan, Tokyo) were placed around the participant’s feet, chair, and electroencephalogram monitor to suppress commercial power noise from the participant’s feet.

Physiological evaluation was performed by recording electroencephalograms (EEG) activity.

### Electroencephalogram (EEG)

**Fig. 16:**
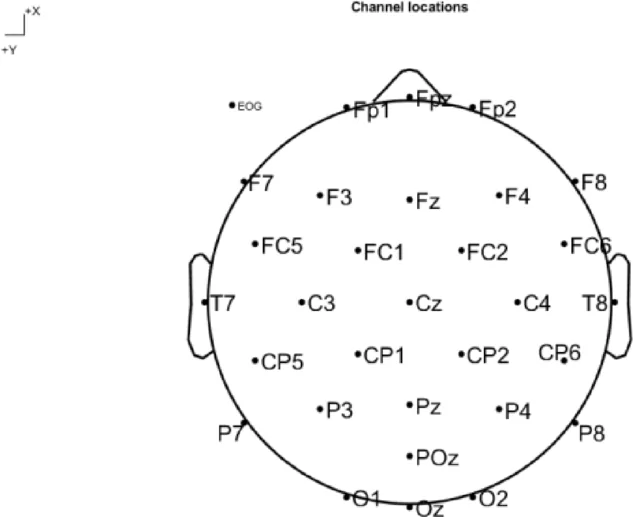
EEG and EOG electrode placement.

EEG signals were recorded using a biological amplifier (Polymate V; Miyuki Giken, Tokyo, Japan) at a sampling frequency of 1,000 Hz. Electrode placement followed the international 10–20 system (Fig. 16). Reference electrodes were placed at M1 (left mastoid) and M2 (right mastoid), and A1 and A2 were connected. Passive electrodes for measuring biological signals with an amplifier were used in this study. The electrodes were fixed on the scalp using paste for measuring bioelectrical signals so that the resistance value was 5 kΩ or less. In this experiment, participants maintained a resting state with their eyes closed during EEG measurements. Brain analysis was performed in MATLAB 2023b^23^ environment with EEG lab library to plot the brain topography and calculate the power spectral density of each brain band, relative power is calculated as shown in Eqs. (1, 2, 3 and 4), together with the calculation of engagement index in Eq. (5). Relative theta band (${R}_{\theta }$) band is calculated from the ratio of frequency range 4 to 8 Hz toward all frequency spectrum, relative Alpha (${R}_{\alpha }$) band is calculated in frequency range between 8 to 13 Hz toward all frequency spectrum, relative beta (${R}_{\beta }$) band is calculated in the frequency range 13 to 30 Hz toward all frequency spectrums. The engagement index (β/(α + θ)) represents the ratio of beta (β) band toward other slow wave (${R}_{\alpha }+{R}_{\theta }$), which correlation with the ability of participant on keep engaging on the task was then calculated based on Eq. (5).

1 $$R_{\theta } = \frac{\theta }{\theta + \alpha + \beta + \gamma }$$

2 $$R_{\alpha } = \frac{\alpha }{\theta + \alpha + \beta + \gamma }$$

3 $$R_{\beta } = \frac{\beta }{\theta + \alpha + \beta + \gamma }$$

4 $$R_{\gamma } = \frac{\gamma }{\theta + \alpha + \beta + \gamma }$$

5 $${\mathrm{Engagement}}\;{\mathrm{index}} = \frac{{R_{\beta } }}{{\left( {R_{\alpha } + R_{\theta } } \right)}}$$

### Statistical analysis

Regarding group comparisons of before-after differences and changes in Igusa presentation and control (no fragrance presentation) groups, data normality was confirmed using the Shapiro–Wilk test, and paired t was used for comparison pairs where normality could be assumed. A two-tailed test was conducted. For comparison pairs for which normality could not be assumed by the Shapiro–Wilk test, the Wilcoxon signed-rank test was performed. The significance level was set at 5% using a two-tailed test. The primary statistical analyses were conducted using JASP^24^. The EEG processing and feature extraction were conducted in MATLAB R2023b with EEGLAB, and GC–MS data handling was performed in MassHunter (Agilent).

## Supplementary Information

Below is the link to the electronic supplementary material.

Supplementary Material 1

## Data Availability

The data sets used and/or analyzed in this study are available from the first author or the corresponding author upon reasonable request.
