# Supplementary material for: Stress resilience and engagement enhancement under the usage of Igusa (Juncus effusus L. var. decipiens Buchen.) mat: a crossover study
Source: Sci Rep. 2026 Sep 8;16:27228. doi: 10.1038/s41598-026-47520-8 (PMC13554183; doi:10.1038/s41598-026-47520-8)
Supplement: Supplementary file 1 [file 41598_2026_47520_MOESM1_ESM.docx]

Supplementary Table S1. Age distribution of participants included in the study.

| Participant ID | Sex | Age (years) |
| --- | --- | --- |
| 2 | Female | 21 |
| 3 | Female | 22 |
| 4 | Female | 22 |
| 5 | Male | 24 |
| 6 | Male | 19 |
| 7 | Male | 23 |
| 8 | Male | 19 |
| 9 | Male | 23 |
| 10 | Female | 18 |
| 11 | Male | 22 |
| 12 | Male | 25 |
| 13 | Female | 21 |
| 14 | Male | 22 |
| 15 | Female | 19 |
| 17 | Male | 23 |
| 18 | Male | 21 |
| 20 | Female | 22 |

(*N*=17).
